# Fee-based open-access journals: A fertile or slippery ground?

**DOI:** 10.4103/0019-5545.74321

**Published:** 2010

**Authors:** R. D. Pattanayak, R. Sagar

**Affiliations:** Department of Psychiatry, All India Institute of Medical Sciences, Ansari Nagar, New Delhi, India - 110 029. E-mail: drraman@hotmail.com.

Sir,

An open-access model is a relative recent concept in academic publishing. In contrast to the subscription model, its full potential is yet to be explored, especially by Indian psychiatrists. In brief, fee-based open access are the online journals for which users are granted an irrevocable, world-wide, perpetual access without any financial or other barriers and the authors (or the sponsoring Institute) bear the cost involved in making it publicly accessible.[1] The peer-review process is similar, albeit much faster, than the traditional scientific journals. The pros (free access, enhanced visibility) and cons (quality concerns) of open-access have been debated.

However, the issue we want to raise is: Are the researchers from developing world likely to derive an overall benefit from an open-access model based on pay for publication? How does this trend affect mental health research from India in the long run?

Over 85% of the world’s population lives in the 153 countries categorized as low and middle income, which generates only 2% of the published mental health research. The research gap has been well documented.[2,3] The costs involved in the open-access journals are too steep to be comfortably affordable (usual charges are 800$-2000$ per article) in developing countries. Further, there is a severe limitation in the number of Institutes or third parties sponsoring such publication costs. In the current list of biocentral members to sponsor publishing, there are 124 members from United States of America compared to mere 6 from India, that too across all biological sciences.[4]

Unlike the print issues, fee-based open-access journals have no definite limit on the number of published articles. It overcomes the space constraints of a print journal but leads to a great increase in the average number of publications per year, most of which are likely to come only from the high income countries. Quite possibly, the gap in published research from developed and developing countries will further widen. The published literature is instrumental in shaping the future of psychiatry and bringing a change. How much will we contribute toward that change in the coming future?

Along with the caution, there is some hope too. The free online availability of research articles may introduce new ideas or themes to undertake research studies and could fuel more research. The recent availability of no-fee open access for several Indian journals, including *Indian Journal of Psychiatry*, is a welcome step and likely to bridge some of the gap. In several parts of India, the institutes or colleges lack infrastructure/funding for library, resulting in unavailability of latest issues or common journals. With open access, all you need is an internet access. May be, in future, we will have indigenous, affordable fee-based open-access journals or more avenues for sponsorships or Institute support. With an ever-increasing numbers of fee-based open-access journals across the world, only time will tell if we would be able to grow with the trend or slip further behind? Hope not.
